# Effect of Educational Intervention Based on Theory of Planned Behavior on Reducing Smoking and Hookah Use Among High School Male Students

**DOI:** 10.1111/crj.70119

**Published:** 2025-08-11

**Authors:** Ali Zarei, Abbas Shamsalinia, Asiyeh Yari, Pooyan Afzali Hasirini, Ali Khani Jeihooni

**Affiliations:** ^1^ Department of Physiology, Estahban School of Paramedical Sciences, School of Nursing Hazrat Zahra (PBUH) Abadeh Shiraz University of Medical Sciences Shiraz Iran; ^2^ Department of Nursing, Nursing Care Research Center Babol University of Medical Sciences Babol Mazandaran Iran; ^3^ Department of Health Education and Health Promotion, School of Health Hormozgan University of Medical Sciences Bandar Abbas Iran; ^4^ Department of Public Health, School of Health Kermanshah University of Medical Sciences Kermanshah Iran; ^5^ Nutrition Research Center, Department of Public Health, School of Health Shiraz University of Medical Sciences Shiraz Iran

**Keywords:** hookah, smoking, students, theory of planned behavior (TPB)

## Abstract

**Background:**

Using smoking and hookah has increased among high school students in recent years. Therefore, the present study aimed to determine the effect of educational intervention based on the theory of planned behaviour (TPB) on reducing smoking and hookah use among high school students.

**Methods:**

This experimental study was conducted on 300 high school male students in Fasa City, Fars Province, Iran, in 2021–2022. Subjects were selected using a simple sampling method and were randomly divided into intervention (*n* = 150) and control (*n* = 150) groups. The educational intervention for the experimental group included 7 sessions of 45–55 min using small group discussion, question and answer, practical demonstrations, video clips, PowerPoint, and booklets. Before the intervention and 3 months after the educational intervention, both experimental and control groups completed the questionnaire. Data were analyzed using SPSS 22 software through Chi‐square, independent *t*‐test, paired *t*‐test, and McNemar test.

**Results:**

The mean age of the experimental and control groups was 17.89 + 1.46 and 17.1 + 1.58 years. The results showed that before the educational intervention, there was no significant difference between the experimental and control groups in terms of awareness, attitude, subjective norms, perceived behavioral control, and behavioral intention; however, 4 months after the educational intervention, there was a significant increase in the experimental group. Also, before the educational intervention, there was no significant difference between the two groups in terms of current smoking and hookah use; however, 4 months after the intervention, there was a significant difference between the two groups.

**Conclusion:**

Implementing the TPB‐directed instructional sessions resulted in reducing smoking and hookah use among high school students.

## Graphical abstract

This experimental study was conducted on 300 high school male students. The educational intervention for the experimental group included 7 sessions. There was no significant difference between the experimental and control groups in terms of awareness, attitude, subjective norms, perceived behavioral control, and behavioral intention; however, 4 months after the educational intervention, there was a significant increase in the experimental group. Implementing the TPB‐directed instructional sessions resulted in reducing smoking and hookah use among high school students.

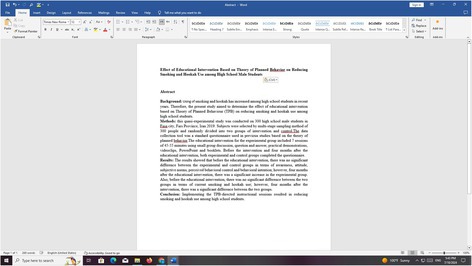

## 1 Introduction

Smoking is one of the major risk factors for cancer, stroke, and respiratory and cardiovascular diseases. Apart from increasing the risk of physical and mental damages, it leads to drug addiction [1]. Smoking is also one of the most dangerous behaviors that disrupt human health; despite many preventive efforts, it is still recognized as one of the most important causes of death in the world [2]. Cigarette smoke has a complex composition with more than 4000 different toxins. Smoking alone causes 90% of lung cancers, 80% of chronic bronchitis, and 25% of other lung diseases. None of the organs in the body are safe from the harmful effects of smoking [3]. Causing death in various ways, smoking is one of the eight leading causes of death in the world. There are currently around one billion people who smoke in the world, and it is estimated that by 2030 another billion young adults will start smoking [4].

The results of the latest study indicate the onset of smoking in adolescence and young adulthood, emphasizing the role of prevention and control measures in this age group [5]. In the last 30 years, the rate of smoking among adults has decreased, whereas the prevalence of smoking among young people has increased [6]. In the United States in 2015, 25.3% of high school students were regular users of any tobacco product [7]. In a study conducted in Iran, the prevalence of smoking in adolescents was reported to be 15.6%. The prevalence of smoking in adolescence is a valuable indicator for predicting the future status of smoking‐related injuries and is important for health policy and planning [8, 9]. About 100 million people around the world use hookah every day. In some places, it is even more prevalent than cigarettes [10]. The number of people who were using hookah weekly in the world is increasing, and the highest prevalence of hookah use occurs in the Middle East and Africa [11]. According to the results of a systematic review, a study found that the prevalence of hookah use was 6% in Pakistan, 11% in Australia, 9%–12% in Syria, 15% in Lebanon, and 11%–15% in Egypt [12]. In Iran, the prevalence of smoking is 17.5%, and hookah is 6.3% [13, 14].

In this regard, the results of a study of Poyrazoğlu et al. indicate the prevalence of hookah use among 32.7% of students [15]. In a study by Taraghi Jah et al., the prevalence of smoking and hookah use among students has been reported as 30.8% and 40.3%, respectively [16, 17].

In many studies, the motivation and causes of smoking in adolescents have been studied, and the effect of many different psychological, behavioral, and demographic risk factors has been investigated, including ethnicity, family structure, parents' socioeconomic status, personal income, parents' smoking, parents' attitudes, family environment, attachment to family and friends, school factors, risky behaviors, lifestyle, depression, unhappiness, self‐esteem, pressure from friends, reducing stress, curiosity, gaining new experiences and trying, pleasure and entertainment, ignorance, and inexperience [18, 19]. Due to the decreasing age of onset, the need for educational programs to raise awareness about the effects and side effects of smoking is strongly felt. Smoking and drug use in young people have potentially many negative consequences for health and well‐being, including increased risk due to interpersonal violence, road accidents, an increased likelihood of engaging in risky sexual behaviors, and an increased risk of suicidal behaviors [20]. Therefore, it is important to address this health problem.

Education is one of the manifestations of empowerment, and its driving force is of great importance [21]. Obviously, educating people about the addictive quality of cigarettes plays a key role in this regard. Considering that changing adolescents' attitudes toward smoking is the first step in the process of preventing addictive behaviors, it is important to provide necessary instruction to students. Education leads to a lasting change in attitude, performance, and ultimately a change in lifestyle [22].

In substance abuse research, it is important to know how cognitive factors such as subjective norms and beliefs are responsible for predicting intention and outcome behavior. In addition, psychosocial factors such as attitude, mental norms, and perceived behavioral control seem to be important in predicting acceptance or rejection of health behaviors [23]. One of the most practical models to predict behavior is the theory of planned behavior (TPB), which could be used to examine the ideas, values, and attitudes within the behavior. The theory is made up of attitude, subjective norms, perceived behavioral control, intention, and behavior, and the occurrence of a particular behavior is conditional on the intention of the individual. In the same vein, the results of similar studies on smoking also indicate the effectiveness of the TPB in predicting and reducing smoking behavior among adolescents [24, 25, 26].

In a snow study et al. in 2018, the results showed that the TPB can have a significant impact on improving smoking behavior in men [3].

Although there is some plausible evidence for the effectiveness of the theory in predicting addictive behaviors, extensive studies are highly recommended to determine its role in preventing risky behaviors. This study was conducted with the aim of investigating the effect of an educational intervention based on the TPB in reducing the use of cigarettes and hookah in Iranian students.

## 2 Materials and Methods

This experimental study was conducted on 300 high school male students (aged 15–19) in Fasa City, in 2021–2022. Recent statistics on the risk factors for non‐communicable diseases have shown that 11.9% of the population aged 15–64 years, most of whom are young boys, consume tobacco on a daily basis. Therefore, the target group of the study was schoolboys.

**FIGURE 1:**
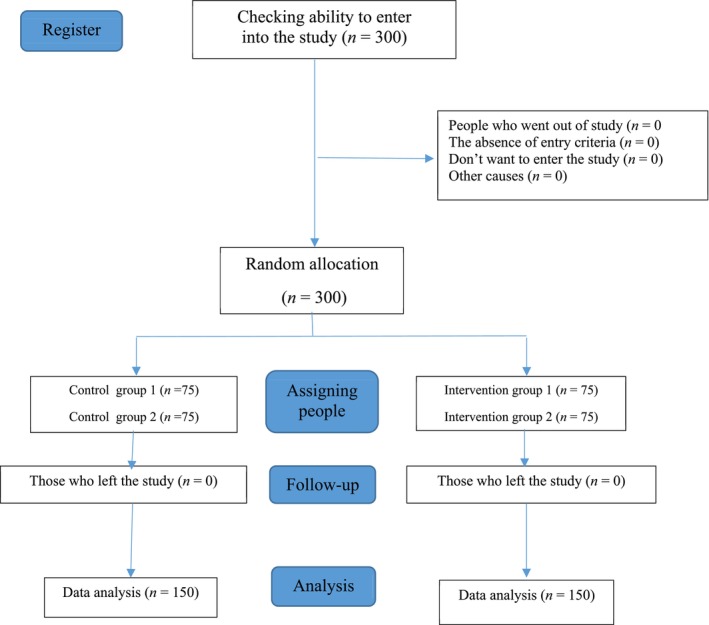
Study population selection chart.

In this study, a multistage sampling method was applied; among 7 boys' high schools in Fasa City, 4 high schools were selected. Thus, in the first stage, according to the number of students in each high school by the stratified sampling method, for each high school (75 persons), the sample ratio required to participate in the study was determined. In the second stage, according to the number of students in the first to third grades, the proportion of the sample participating in the study from each class was determined. In the third step, the required number of samples from each class was selected by simple random sampling from the attendance list (Figure 1). Inclusion criteria were male high school students aged 15–19, willingness to participate in the study, and having a history of smoking at least once a week. Exclusion criteria were unwillingness to participate in the study. The sample size was determined based on a review of similar studies [27, 28]. The mean behavior change in the intervention group was 1.3 before the intervention with a standard deviation of 0.37 and after the intervention 1.48 with a standard deviation of 0.25 according to study number 19. With a 95% confidence level and 90% power, the sample size was calculated for each group as 65 people, and 75 people were selected in each group to increase power and compensate for possible loss.

## 3 Ethical Consideration

Although obtaining permission from the ethics committee of Fasa University of Medical Sciences and the Fasa Department of Education, the goals, importance, and necessity of the research project were explained to the participants. Also, after obtaining written consent from parents and students, the participants were assured that their information would be completely confidential.

## 4 Data Collection Tools

Data were collected using written questionnaires and the self‐report method. The questionnaire consists of two components, including demographic characteristics (age, parents' education, parents' occupation, history of tobacco use, and history of tobacco use by relatives and friends). The questionnaire was based on studies by Fathi et al. [26], Karimy et al. [24], and Abedin et al. [29]. The overall reliability of the tool was 0.89. The Cronbach's alpha of subjective norms, perceived behavioral control, behavioral intention, and awareness was 0.92, 0.78, 0.74, 0.82, and 0.92, respectively. Awareness of the harms of smoking was measured by 15 multiple‐choice questions, scored between 0 and 15. Attitude toward smoking was measured by 8 questions based on a 5‐point Likert scale. The answers ranged from *strongly disagree* (scored 1) to *strongly agree* (scored 7). Subjective norms encouraging tobacco use were measured by 8 questions based on a 5‐point Likert scale. The answers ranged from *strongly disagree* (scored 1) to *strongly agree* (scored 7).

Perceived behavioral control was measured by 8 questions based on a 5‐point Likert scale. The answers ranged from *strongly disagree* (scored 1) to *strongly agree* (scored 7). Behavioral intention was also measured by yes/no questions (*yes* = 1, *no* = 0).

## 5 Educational Intervention

The educational intervention for the experimental group consisted of 7 sessions of 45–55 min using small group discussion, question and answer, practical demonstration, video clips, PowerPoint, and pamphlet, which was held once a week (Table 1). The control and review of the questionnaire and the implementation of educational interventions were carried out by noncommunicable disease experts, one person with a doctorate in psychology and one person with a doctorate in health education and health promotion.

**TABLE 1:** Educational intervention sessions.

Training session	Educational content
First and second	Introduction and objectives, risks of smoking and hookah
Third	Attitudes toward smoking and hookah
Fourth and fifth	The effect of peers and family on smoking and hookah
Sixth and seventh	Control behavior planned to change behavior, browse content, and clear up ambiguities
Learning assist tools	Small group discussion, question and answer, practical demonstrations, videoclips, PowerPoint, and booklets

In these sessions, the consequences of smoking and hookah use, prevention of smoking and hookah use, improving problem‐solving skills, promoting self‐control behaviors and saying no, implementing a smoking cessation solution, increasing a positive attitude toward avoiding smoking and hookah use, and increasing students' ability to quit smoking and hookah use were discussed. A session was also held with teachers, school officials, a family member, and staff of health centers as subjective norms. The experimental group was divided into 5 groups of 30, and the role of friends to quit smoking and hookah use was emphasized.

At the end of the training sessions, an educational booklet on the effects of tobacco use and problem‐solving skills was provided. A WhatsApp group was also formed to send educational and motivational messages and exchange information. To follow up the activities, a training session was held for the students every month. The experimental and control groups completed the questionnaire before and 4 months after the completion of the educational intervention. At the end of the study, according to ethical considerations, a meeting was held for the control group, and educational content was provided to them.

## 6 Data Analysis

The collected data were analyzed in SPSS 22 software using the Kolmogorov–Smirnov test, chi‐square test, independent *t*‐test, McNemar test, and paired *t*‐test. The variables of awareness, attitude, perceived behavioral control, subjective norms, and intention were independent variables, and behavior was the dependent variable (*p* = 0.05).

## 7 Results

In this study, 300 high school male students participated. The mean age of the experimental and control groups was 17.89 + 1.46 and 17.68 + 1.58 years, respectively. Based on the independent *t*‐test, no significant difference was observed between the two groups (*p* = 0.327). Based on the chi‐square test, there was no significant difference between the two groups in terms of smoking history (*p* = 0.242), history of hookah use (*p* = 0.355), mothers' occupation (*p* = 0.206), fathers' occupation (*p* = 0.194), fathers' education (*p* = 0.192), mothers' education (*p* = 0.184), history of smoking by relatives (*p* = 0.249), and history of hookah use by relatives (*p* = 0.173) (Table 2).

**TABLE 2:** Comparison of frequency distribution of demographic variables between experimental and control groups.

Variable		Experimental group		Control group		*p*
		Number	Percentage	Number	Percentage	
History of smoking	Yes	86	57.33	81	54	0.292
	No	64	42.67	69	46	
History of hookah use	Yes	63	42	66	44	0.355
	No	87	58	84	56	
Mother's occupation	Housewife	90	60	97	64.67	0.206
	Employed	28	18.67	35	23.33	
	Self‐employed	6	4	2	1.33	
	Other	26	17.33	16	10.67	
Father's occupation	Unemployed	18	19.33	21	14	0.194
	Employed	75	34	64	42.67	
	Self‐employed	42	25.34	38	25.33	
	Other	15	21.33	27	18	
Father's education	Illiterate	0	0	0	0	0.192
	Elementary school	22	14.66	24	16	
	Secondary school	28	18.67	34	22.67	
	High school	64	42.67	70	46.67	
	Academic	36	24	22	14.66	
Mother's education	Illiterate	1	0.67	1	0.67	0.184
	Elementary school	20	13.33	29	19.33	
	Secondary school	48	32	50	33.34	
	High school	54	36	48	32	
	Academic	27	18	22	14.66	
History of smoking by relatives	Yes	94	62.67	86	57.33	0.249
	No	56	37.33	64	42.67	
History of hookah use by relatives	Yes	84	56	72	48	0.173
	No	66	44	78	52	

The results showed that before the educational intervention, there was no significant difference between the experimental and control groups in terms of awareness, attitude, subjective norms, perceived behavioral control, and behavioral intention; however, 4 months after the educational intervention, there was a significant increase in the amount of these variables in the experimental group (Table 3).

**TABLE 3:** Comparison of mean scores of awareness, attitude, subjective norms, perceived behavioral control, and behavioral intention in the experimental and control groups before and 4 months after the educational intervention.

Variables	Group	Before the intervention	4 months after the intervention	*p*
Awareness	Experimental	7.1 ± 22.12	13.1 ± 4.27	0.001
	Control	7.1 ± 13.16	7.1 ± 20.14	0.262
	*p*	0.231	0.001	
Attitude	Experimental	18.3 ± 34.10	46.3 ± 25.47	0.001
	Control	20.3 ± 15.03	23.3 ± 27.11	0.204
	*p*	0.202	0.001	
Perceived behavioral control	Experimental	20.3 ± 14.09	41.3 ± 18.30	0.001
	Control	19.3 ± 19.42	20.3 ± 7.40	0.274
	*p*	0.312	0.001	
Subjective norms	Experimental	16.3 ± 55.11	47.3 ± 14.23	0.001
	Control	18.3 ± 22.07	20.3 ± 42.11	0.199
	*p*	0.214	0.001	
Intention	Experimental	13.2 ± 68.72	37.2 ± 26.40	0.001
	Control	15.2 ± 98.75	18.2 ± 02.69	0.239
	*p*	0.203	0.001	

Also, before the educational intervention, there was no significant difference between the experimental and control groups in terms of current smoking and hookah use. But 4 months after the intervention, there was a significant difference between the two groups (Table 4).

**TABLE 4:** Comparison of current tobacco use before and 4 months after educational intervention in experimental and control groups.

Variables	Group	Before the intervention		4 months after the intervention		*p*
		Number	Percent	Number	Percent	
Smoking	Experimental	86	57.33	41	27.33	0.001
	Control	77	51.33	77	51.33	
	*p*	0.280		0.001		
Hookah use	Experimental	81	54	52	34.67	0.001
	Control	88	58.68	88	58.67	
	*p*	0.289		0.001		

## 8 Discussion

The present study was conducted to determine the effectiveness of educational intervention based on the TPB in reducing smoking and hookah use among high school students.

The results of this study showed that the mean score of awareness in the experimental group significantly increased after the intervention, indicating the effectiveness of the educational program based on the TPB in improving negative attitudes toward smoking. This is consistent with the results of studies by Levine et al. [30] and Nebhinani et al. [31]. Consistent with the present study, Levin et al. [30] and Bastami [32] emphasized the role of knowledge and attitude in the tendency toward addiction. The first step is to identify and dispel these misconceptions. If the illusion that life without smoking would be no longer enjoyable is discredited, people who smoke will quit easily. The role of education in raising awareness and eliminating misconceptions is undeniable; therefore, if the necessary knowledge on the harmful effects of smoking and hookah use is provided for students of different ages, especially in school, they will be less inclined to smoke. Playing a major role in raising awareness as a tool of social control, education could prevent and reduce the occurrence of smoking.

According to the results, in terms of attitude, the experimental group has a statistically significant increase compared to the control group, indicating the effectiveness of the training program based on the TPB. This is consistent with the results of studies by Fathi et al. [26], Mahmoud et al. [33], and Barati et al. [34]. In our society, smoking, especially by adolescents and students, is against the social norms and cultural beliefs. Not only is smoking not a privilege and indicator of superiority, but the majority of people in society have a negative attitude toward people who smoke, and in many cases, the reasons for the tendency to use nicotine and cigarettes can be a serious ground for the tendency to all kinds of addictions, so smoking is considered a gateway to drug addiction [1]. Unfortunately, today, cigarettes are easily accessible without any restrictions. On the other hand, many people who enjoy great popularity start smoking, which encourages adolescents to smoke. Life skills training enables people to resolve their conflicts with peers in a constructive way; their ability to control impulses increases, and increasing their cognitive coping skills reduces their tendency to use drugs and prevents smoking [35]. Therefore, the training program of building healthy behaviors can negatively affect the attitudes of participants in various dimensions.

In the present study, the mean score of subjective norms after the educational intervention in the experimental group was significantly higher than that of the control group. The results of studies by Jalambadani et al. [36], Hoseini Soorand et al. [37], Larki et al. [38], and Fathi et al. [26] are consistent with the results of the present study. Subjective norm is the perception of social pressure to do or not to perform a risky behavior, which is expressed under the influence of perceived social pressure, and its value depends on the motivation of the individual to meet the expectations of others. The results of the present study are consistent with the results of studies by Jadgal et al. [39] and Bashirian et al. [4]. Smoking is also learned through symbolic interaction in small groups. For the first time, novices learn to stimulate smoking. These behaviors and experiences are considered acceptable and enjoyable by many peers, so there is a possibility of continued consumption [40]. In the study by Dadipoor et al. [41], with an increase of one score in attitude, the probability of quitting smoking increased by 31%; with an increase of one score in knowledge, the probability of quitting smoking increased by 0.05%. With an increase of one score in intention, the probability of quitting smoking increased by 26%. In social norms, the probability of quitting smoking increased by 0.02%; with an increase of one score in perceived control, the probability of quitting smoking increased by 16%. Due to the fact that students are influenced by the beliefs of friends, family, and other important ones in life, it is highly recommended to take the initiative to design and implement educational interventions aimed at decreasing subjective norms encouraging tobacco use.

The results of this study showed that the mean score of perceived behavioral control after the intervention was significantly higher in the experimental group compared to the control group, which is consistent with the results of similar studies [42, 43]. According to the TPB, perceived behavioral control is one's awareness of the fact that to what extent to/not to perform a given behavior is under their conscious control. People are more likely not to smoke who believe that smoking cessation is under their control. Also, in social psychology studies, the level of behavioral control in people with poor self‐esteem is reported to be low. This is consistent with the results of the studies by Alanazi et al. [44]; subjective norm through attitude and perceived behavioral control had a statistically significant indirect effect on intention to smoke.

The results of the present study also showed that the mean score of behavioral intention in the experimental group increased significantly after the intervention, which is consistent with the results of similar studies [33, 43]. Based on the results of the present study, smoking and hookah use in the intervention group decreased significantly after the training program compared to the control group, indicating the effectiveness of the educational program based on the TPB. This is consistent with the results of the studies by Tavousi et al. [33] and Karimy et al. [24]. In the study by Bashirian et al. [45], the constructs of attitude, subjective norms, and intention were effective in reducing hookah smoking behavior in adolescent girls in the intervention group. Also, in the study of Shahriyarimoghadam et al. [46], after the educational campaign, there was a significant increase in the scores of the TPB constructs (attitude, subjective norms, perceived behavioral control, and behavioral intention) and a reduction in hookah smoking. In the study by Ghasemian et al. [47], the educational intervention had significant effects on increasing knowledge, reducing tobacco use, increasing intention, attitude, subjective norm, and perceived behavioral control.

Parents can play a significant role in how their children cope with stressful issues and also reduce their behavioral problems, as it has been shown that the level of education of parents has an impact on their children's social skills, especially by having a successful and stable relationship with their peers. Additionally, increasing the level of mother's education increases social behaviors. There is a significant relationship between behavioral problems and mother's level of education. However, in the present study, no significant relationship was observed with the tendency of people to smoke and with the level of education and occupation of parents [48]. In the study by Shubayr et al. [49], negative attitudes toward tobacco were inversely related to tobacco use among students. Subjective norms, which reflect perceived social pressure, were positively related to tobacco use. Perceived behavioral control, which reflects the ease of quitting or avoiding tobacco, was strongly associated with tobacco use. Also, stronger behavioral tendencies to use tobacco were associated with a higher likelihood of use.

Despite the positive points, the present study has limitations: the evaluation period of the outcomes was only 4 months after the intervention, which can be extended in future research in order to better evaluate the outcomes. Examining smoking status by self‐reporting of students is not generalizable. Participants in the experimental condition probably knew what the authors wanted their responses to be next. It is difficult to know which specific components of this multicomponent program had the greatest impact.

## 9 Conclusion

Considering the prevalence of smoking and hookah use among students, educational programs to prevent smoking and hookah use and reduce its consumption based on the TPB can be effective. Learning health knowledge, attitudes, and behaviors begin at an early age. Thus, performing programs for prevention of smoking and hookah in schools on the basis of models whose efficiency has been well proved, such as TPB, at an early age and repeating the trainings in the adolescence period play an important role in prevention of smoking and hookah. Future researchers can take advantage of the TPB when designing training materials and performing training interventions in order to identify and design a framework of interventions.

## Author Contributions

A.Z., A.S., A.Y., P.A.H., and A.K.J. assisted in the conceptualization and design of the study, oversaw data collection, conducted data analysis, and drafted the manuscript. A.K.J. and A.Y. conceptualized and designed the study, assisted in data analysis, and reviewed the manuscript. A.Z., A.S., A.Y., P.A.H., and A.K.J. assisted in study conceptualization and reviewed the manuscript. All authors read and approved the final manuscript.

## Ethics Statement

Ethical approval was obtained from the Human Research Ethics Committee at the Fasa University of Medical Sciences (IR.FUMS.REC.1396.305). Informed consent was taken from all the participants. For students involved, informed consent from a parent and/or legal guardian was obtained in the study. All methods were carried out in accordance with the declarations of Helsinki. There was an emphasis on maintaining privacy in keeping and delivering the information accurately without mentioning the names of the participants. The participants were given the right to leave the interview at any time, and they were promised access to the study results.

## Consent

The authors have nothing to report.

## Conflicts of Interest

The authors declare no conflicts of interest.

## Data Availability

The datasets used and/or analyzed during the current study are available from the corresponding author on reasonable request.
